# Prediction Model of Clearance by a Novel Quantitative Structure–Activity Relationship Approach, Combination DeepSnap-Deep Learning and Conventional Machine Learning

**DOI:** 10.1021/acsomega.1c03689

**Published:** 2021-09-01

**Authors:** Hideaki Mamada, Yukihiro Nomura, Yoshihiro Uesawa

**Affiliations:** † Department of Medical Molecular Informatics, Meiji Pharmaceutical University, 2-522-1, Noshio, Kiyose-shi, Tokyo 204-858, Japan; ‡ Drug Metabolism and Pharmacokinetics Research Laboratories, Central Pharmaceutical Research Institute, Japan Tobacco Inc., 1-1, Murasaki-cho, Takatsuki, Osaka 569-1125, Japan

## Abstract

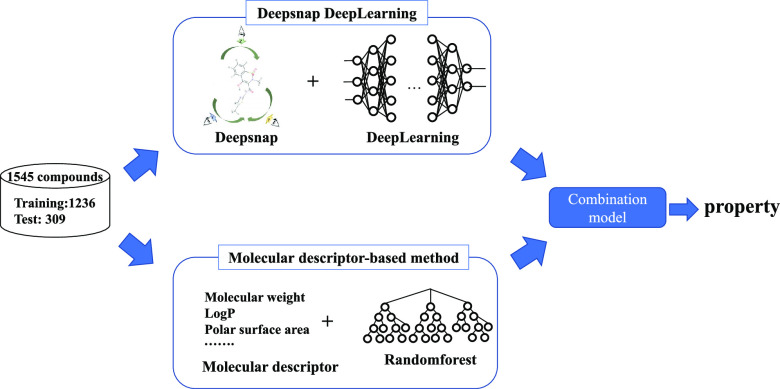

Some targets predicted by machine learning (ML) in drug discovery remain a challenge because of poor prediction. In this study, a new prediction model was developed and rat clearance (CL) was selected as a target because it is difficult to predict. A classification model was constructed using 1545 in-house compounds with rat CL data. The molecular descriptors calculated by Molecular Operating Environment (MOE), alvaDesc, and ADMET Predictor software were used to construct the prediction model. In conventional ML using 100 descriptors and random forest selected by DataRobot, the area under the curve (AUC) and accuracy (ACC) were 0.883 and 0.825, respectively. Conversely, the prediction model using DeepSnap and Deep Learning (DeepSnap-DL) with compound features as images had AUC and ACC of 0.905 and 0.832, respectively. We combined the two models (conventional ML and DeepSnap-DL) to develop a novel prediction model. Using the ensemble model with the mean of the predicted probabilities from each model improved the evaluation metrics (AUC = 0.943 and ACC = 0.874). In addition, a consensus model using the results of the agreement between classifications had an increased ACC (0.959). These combination models with a high level of predictive performance can be applied to rat CL as well as other pharmacokinetic parameters, pharmacological activity, and toxicity prediction. Therefore, these models will aid in the design of more rational compounds for the development of drugs.

## Introduction

Quantitative structure–activity relationship (QSAR) analysis is a method to predict the absorption, distribution, metabolism, and excretion (ADME) parameters of small-molecule compounds based on their molecular structure. QSAR is used to predict ADME parameters including solubility,^1^ protein binding,^2^ permeability,^3^ blood-to-plasma concentration ratios,^4^ and metabolic stability,^5^ as well as *in vivo* pharmacokinetic (PK) parameters [clearance (CL), volume of distribution (Vd), and half-life].^6,7^ The construction of QSAR models has mostly used molecular descriptors and fingerprints as features of compounds, as well as multiple regression, partial least-squares regression, random forest, support vector machines, neural networks, and XGBoost as algorithms. However, some prediction methods that use conventional ML for ADME parameters have a poor prediction accuracy.

Recently, applying Deep Learning (DL) to the prediction of ADME parameters demonstrated accuracy improvements over other conventional ML methods.^8−10^ In these reports, molecular descriptors and fingerprints were used for DL as features of the compounds. However, the use of new features is expected to further improve the prediction accuracy in addition to DL. Uesawa recently reported a new method called DeepSnap, which uses images of compounds as features for DL.^11^ DeepSnap and Deep Learning (DeepSnap-DL) provided better predictions of toxicological targets including mitochondrial membrane potential disruption, constitutive androstane receptor (CAR), and aryl hydrocarbon receptor (AhR) compared with conventional ML.^11−17^ However, there have been no reports on ADME parameters using DeepSnap-DL. Therefore, constructing ADME parameters using DeepSnap-DL might have good prediction accuracy similar to that for toxicological targets.

Among ADME parameters, CL is an important PK parameter for drug discovery. CL in animal species, such as rats, is used to understand the relationship between compound exposure in animals and humans regarding PK, toxicity, and drug effects. It is desirable to obtain compounds that have an acceptable PK profile. However, most compounds do not have an acceptable PK profile at the early drug discovery stage. Grime *et al*. reported the efficient and cost-effective pursuit of candidate compounds with acceptable PK profiles.^18^ Pharmaceutical companies typically perform PK experiments in rats, including intravenous and oral administration, to determine whether compounds have an acceptable profile. To reduce the overall drug discovery cost, time, and animal usage, it is ideal to predict the rat PK profile before new chemical synthesis. QSAR can make predictions at early stages of drug development, even for virtual compounds, and can therefore help in the rational design of drug compounds.

Muegge *et al*. and McIntyre *et al*. reported QSAR models of rat clearance based on 6000–17,529 expanded in-house compounds.^19,20^ They constructed rat CL models using conventional ML. In their models, molecular descriptors and fingerprints were used as features of compounds. The naïve Bayesian method used by McIntyre *et al*. and random forest or support vector machines (specific machine details not provided in Muegge *et al*.) were used as algorithms. However, these prediction models did not show sufficient prediction performance [accuracy (ACC) of 0.74 and an area under the curve (AUC) of 0.82]. Although the prediction of rat CL is important in drug discovery, it is one of the evaluation targets that are difficult to predict by conventional ML. Therefore, in this study, we selected rat CL prediction as a difficult prediction target in drug discovery and developed a new prediction model using combination DeepSnap-DL and conventional ML to improve the evaluation metrics.

## Results

### Separation of Compounds into Training and Test Datasets and Their Verification by Chemical Space Analysis

**Figure 1:**
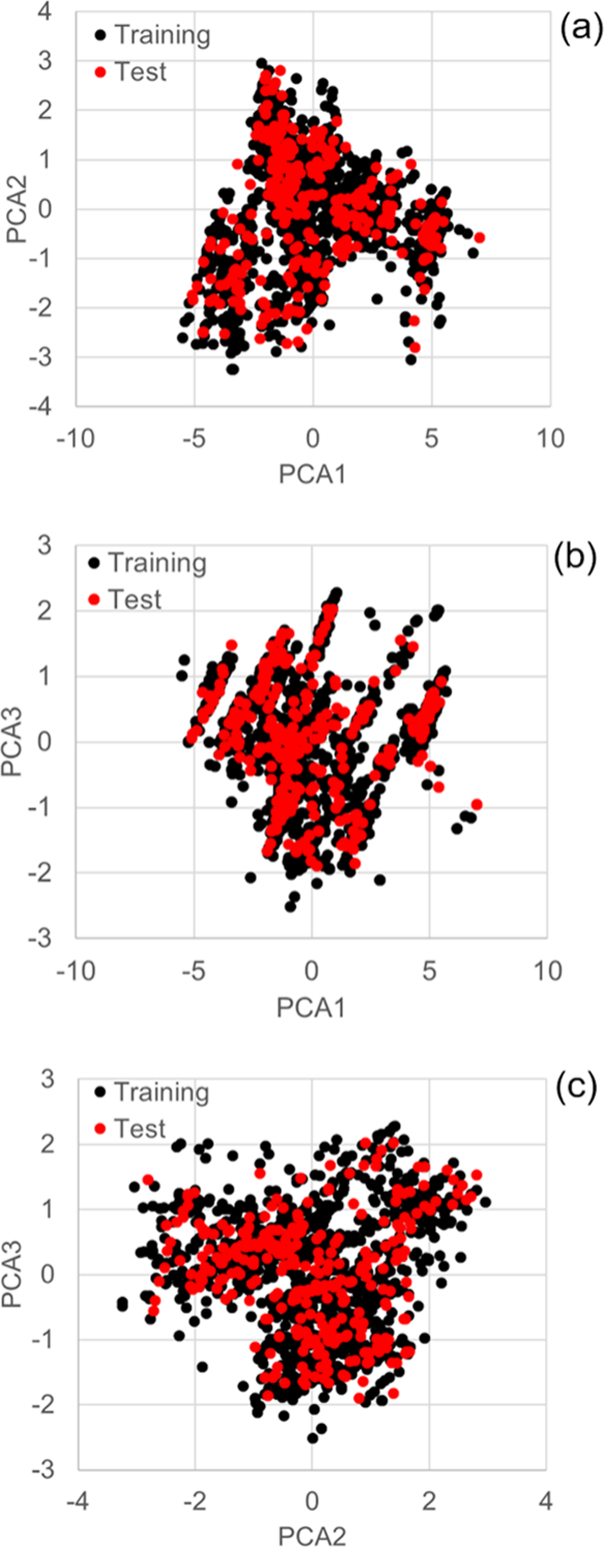
Three-component PCA score plots based on 11 representative molecular descriptors (*n* = 1545). (a) Score plot of PCA1 (62.3%) and PCA2 (12.0%). The horizontal axis is the first principal component and the vertical axis is the second principal component. (b) Score plot of PCA1 (62.3%) and PCA3 (8.01%). The horizontal axis is the first principal component and the vertical axis is the third principal component. (c) Score plot of PCA2 (12.0%) and PCA3 (8.01%). The horizontal axis is the second principal component and the vertical axis is the third principal component. Each dot represents a compound. Black circles indicate the training set (*n* = 1236) and red circles indicate the test set (*n* = 309). PCA, principal component analysis.

Principal component analysis (PCA) was performed using a dataset of 1545 compounds with 11 representative molecular descriptors to confirm the correctness of the compound separation. It was previously reported that PCA could show the distribution of chemical space in the dataset.^21^ Components 1, 2, and 3 explained 62.3, 12.0, and 8.0% of the variance, respectively. Figure 1 shows that the compounds were effectively separated into the training and test datasets.

### Construction of CL Prediction Models Using Molecular Descriptors by DataRobot

The CL prediction models were constructed using 4795 molecular descriptors by DataRobot. First, random forest was selected as the algorithm based on the results of logloss of internal validation. Then, 100 molecular descriptors were selected from 4795 molecular descriptors using the permutation importance of random forest (Table S1). Second, these 100 descriptors were used to build a prediction model. Over 40 prediction models were constructed and evaluated. The top three models are shown in Table 1, and all results are shown in Table S2. Among these models, random forest showed the lowest logloss results. Based on this result, a final prediction model was constructed using 100% training data by random forest. The results of the evaluation metrics for the test datasets are shown in Table 2. AUC, balanced accuracy (BAC), ACC, sensitivity, specificity, F-measure, precision, recall, and Matthews correlation coefficient (MCC) were 0.883, 0.817, 0.825, 0.772, 0.863, 0.784, 0.797, 0.772, and 0.638, respectively.

**Table 1:** Internal Validation Results of the Top Three Models Using Molecular Descriptor-Based Methods^a^

model	logloss
random forest	0.4254
AVG Blender	0.4282
ENET Blender	0.4297

a Logloss, logloss value from 5-fold validation; AVG Blender, average Blender; ENET Blender, Elastic-Net Blender.

**Table 2:** External Test Results^a^

	*n*	AUC	BAC	ACC	sensitivity	specificity	F-measure	precision	recall	MCC
MD-based method	309	0.883	0.817	0.825	0.772	0.863	0.784	0.797	0.772	0.638
DeepSnap-DL	309	0.905	0.833	0.832	0.843	0.824	0.805	0.770	0.843	0.659
ensemble model	309	0.943	0.868	0.874	0.835	0.901	0.845	0.855	0.835	0.739
consensus model	214		0.958	0.959	0.953	0.963	0.948	0.943	0.953	0.915

a MD-based method, molecular descriptor-based method; AUC, area under the curve of the receiver operating characteristic curve; BAC, balanced accuracy; ACC, accuracy; MCC, Matthews correlation coefficient.

### Construction of CL Prediction Models by DeepSnap-DL

The present study was conducted at four angles (65°, 85°, 105°, and 145°), with five learning rate conditions (0.0000001–0.001) and five maximum epoch conditions (15–300). In each condition, the epoch with the lowest loss [DeepSnap (Validation)] was selected as the epoch for calculating the evaluation metrics and the results of AUC [DeepSnap (Training)], AUC [DeepSnap (Validation)], and AUC (test) were calculated, respectively (Table S3). The AUC [DeepSnap (Validation)] was calculated for each condition, and the condition with the highest AUC [DeepSnap (Validation)] was selected as the final model in DeepSnap-DL. The highest AUC [DeepSnap (Validation)] in all conditions was observed at 145°, with a maximum epoch of 300 and a learning rate of 0.000001 (Table 3). At a learning rate of 0.000001 with a maximum epoch of 300, the AUC [DeepSnap (Validation)] results for 105° and 145° were 0.8968 and 0.8974, respectively, which were higher for 145°. In this condition, the results of the test datasets in the final model in DeepSnap-DL are shown in Table 2. AUC, BAC, ACC, sensitivity, specificity, F-measure, precision, recall, and MCC were 0.905, 0.833, 0.832, 0.843, 0.824, 0.805, 0.770, 0.843, and 0.659, respectively.

**Table 3:** AUC of Validation Results by DeepSnap-DL

learning rate	max. epoch	145°	105°	85°	65°
0.001	15	0.812	0.779	0.799	0.500
	30	0.771	0.787	0.798	0.500
	60	0.500	0.789	0.500	0.820
	100	0.500	0.791	0.500	0.788
	300	0.818	0.777	0.500	0.782
0.0001	15	0.850	0.793	0.809	0.790
	30	0.831	0.803	0.797	0.770
	60	0.801	0.840	0.817	0.791
	100	0.801	0.813	0.806	0.791
	300	0.814	0.820	0.842	0.802
0.00001	15	0.879	0.882	0.873	0.831
	30	0.887	0.868	0.833	0.837
	60	0.879	0.824	0.830	0.860
	100	0.868	0.841	0.839	0.855
	300	0.867	0.848	0.837	0.857
0.000001	15	0.740	0.851	0.864	0.875
	30	0.847	0.870	0.883	0.844
	60	0.867	0.884	0.893	0.887
	100	0.877	0.889	0.893	0.879
	300	0.897	0.897	0.891	0.863
0.0000001	15	0.644	0.625	0.670	0.701
	30	0.650	0.661	0.728	0.820
	60	0.666	0.729	0.828	0.856
	100	0.689	0.826	0.850	0.871
	300	0.844	0.869	0.883	0.844

### Ensemble Model with Combination DeepSnap-DL and Conventional ML

The average of the predicted probabilities obtained from conventional ML using the molecular descriptors and DeepSnap-DL was calculated as the new predicted probability (ensemble model). Table 2 shows the results of the evaluation metrics of test sets using the probabilities of these averages. AUC, BAC, ACC, sensitivity, specificity, F-measure, precision, recall, and MCC were 0.943, 0.868, 0.874, 0.835, 0.901, 0.845, 0.855, 0.835, and 0.739, respectively. The evaluation metrics of the ensemble model showed better results than conventional ML using the molecular descriptors and DeepSnap-DL.

### Consensus Model with Combination DeepSnap-DL and Conventional ML

The test results of the confusion matrix of conventional ML using the molecular descriptors and DeepSnap-DL results are shown in Table 4a,b. Based on these results, a consensus model was constructed using the results of the agreement (Table 4c). Evaluation metrics showed that BAC, ACC, sensitivity, specificity, F-measure, precision, recall, and MCC were 0.958, 0.959, 0.953, 0.963, 0.948, 0.943, 0.953, and 0.915, respectively (Table 2). The number of predictable compounds decreased from 309 to 214. However, these results showed that the consensus model was highly accurate for all the evaluation metrics.

**Table 4:** Confusion Matrix for Rat CL Classification^a^

		predicted	
(a) MD-based method		low clearance	high clearance
observed	low clearance	98	29
	high clearance	25	157

		predicted	
(b) DeepSnap-DL		low clearance	high clearance
observed	low clearance	107	20
	high clearance	32	150

		predicted	
(c) consensus model		low clearance	high clearance
observed	low clearance	82	4
	high clearance	5	130

a MD-based method, molecular descriptor-based method; DeepSnap-DL, DeepSnap and Deep Learning; low clearance, CL < 1 L/h/kg; high clearance, CL ≥ 1 L/h/kg.

## Discussion

During drug discovery, prediction models are constructed for various targets such as toxicity and ADME parameters, but the prediction performance of these models is insufficient for some targets. Therefore, a new prediction model that has a high level of predictive performance is desired. In this study, we focused on the prediction of rat CL as an important and difficult prediction target in drug discovery. To improve the prediction performance, we developed a new prediction model with DeepSnap-DL, which uses images for ML.

For rat CL dataset creation, compounds were separated into training and test sets (Table 5 and Figure 1). To ensure unbiased segregation, the PCA analysis was conducted using 11 representative molecular descriptors (Table S4), which are generally considered important for synthetic expansion.^21^ We also examined the distribution of each training set and test set for the 11 descriptors (Figure S1). As shown in Figure 1 and Figure S1, the separation was well balanced and the cumulative contribution ratio of PCA from 1 to 3 was 82.31%.

**Table 5:** Number of Chemical Compounds in Training and Test Datasets^a^

score	training	test	sum
low clearance	509	127	636
high clearance	727	182	909
sum	1236	309	1545

a Low clearance, CL < 1 L/h/kg; high clearance, CL ≥ 1 L/h/kg.

For rat CL prediction, two models have been reported to date, although a direct comparison is difficult because both are different in-house compounds.^19,20^ However, the prediction performance of both models was low, with an ACC of 0.74 and an AUC of 0.82.^19,20^ These models also used molecular descriptors and fingerprints as features of compounds, as well as random forest (or support vector machines) and naïve Bayesian as algorithms. In this study, we used molecular descriptors obtained from three software, Molecular Operating Environment (MOE), alvaDesc, and ADMET Predictor, and constructed a model using DataRobot, which allows multiple algorithms to be considered simultaneously as conventional ML. As a result, evaluation metrics calculated an ACC of 0.825 and an AUC of 0.883 (Table 2). Although it is difficult to make a direct comparison because of the different compounds used, we constructed a prediction model that surpassed previous models by adopting multiple software for molecular descriptors and multiple algorithms.

We developed a prediction model using DeepSnap, which uses images as features of compounds and DL as an algorithm. First, we examined the hyperparameters of DeepSnap-DL for predicting rat CL. The results for all hyperparameter combination conditions in this study are shown in Table S3, and the AUC results for internal validation are shown in Table 3. The condition of 145°, learning rate of 0.000001, and maximum epoch of 300 showed the highest value of AUC [DeepSnap (Validation)] (Table 3). We evaluated the prediction performance of the test sets using this condition for the final model of DeepSnap-DL. As shown in Table 2, the ACC was 0.832 and the AUC was 0.905, which were higher than when using the molecular descriptor-based method (conventional ML). It was reported that DeepSnap-DL had a higher prediction performance than conventional ML for multiple toxicity targets of progesterone receptor, CAR, and AhR.^15−17^ Although DeepSnap-DL has only been used for toxicity targets, it also had high prediction performance for PK parameters.

In this study, we focused on the multiple QSAR model to improve the prediction performance further. The multiple QSAR models [ensemble learning, combinatorial (combi) QSAR, and consensus classification] were used to improve prediction and obtain stable results by combining different features or algorithms.^22−24^ Various multiple QSAR models have been reported to date. Combi QSAR is a method for constructing models by combining molecular descriptors in multiple commercial software and multiple algorithms (*k*-nearest neighbor, support vector machine, decision trees, and random forest).^25−28^ It was reported that high prediction accuracy and stable results were obtained by using these methods.^25−28^ Furthermore, Brownfield *et al*. proposed a prediction method that combined three class systems by a fusion process as a consensus classification.^29^ Kim *et al.* and Wang *et al*. showed an improvement in prediction accuracy by using a molecular descriptor and the parameter of transporter as a biological descriptor for the prediction model.^30,31^ From these findings, it was expected that the use of multiple prediction models and different types of features might improve the prediction performance. Therefore, we investigated the combination of prediction models using a molecular descriptor-based model and DeepSnap-DL. We developed two multiple QSAR models, the ensemble model and consensus model. For the ensemble model, the average of the prediction probabilities obtained from the molecular descriptor-based model and DeepSnap-DL was used. As a result, the AUC and ACC were improved because these scores increased from 0.883–0.905 to 0.943 and from 0.825–0.832 to 0.874, respectively (Table 2). To confirm that this was not a coincidence, we examined different test partitions (Figure S2), which showed that the average of the prediction probabilities improved the prediction performance (Table S5). For the consensus model, only the results that agreed with the molecular descriptor-based method and DeepSnap-DL were used. The evaluated number of compounds decreased from 309 to 214 because different prediction results could not be used. However, the accuracy of prediction using the consensus model was improved from 0.825–0.832 to 0.959 (Table 2). We examined different test partitions as was done for the ensemble model and found that using the consensus model improved the prediction performance (Figure S2 and Table S5). Although the consensus model has been shown to have the highest prediction accuracy, it is not possible to evaluate all test compounds. In fact, the number of compounds that can be evaluated by the consensus model has been reduced to 214–226 (Table 2 and Table S5). In the early stages of drug screening, a large number of compounds need to be evaluated comprehensively without omission. The model that can evaluate all compounds is suitable, so further efforts are needed for practical application. In this study, the ensemble model and the consensus model improved the prediction performance. To the best of our knowledge, this is the first report showing an improvement in prediction performance using images and molecular descriptors of compounds. The reason for the improved prediction performance using this combination is that the recognition of compounds in the image space and molecular descriptor space is different. This suggests that the high prediction model was achieved by using information in each space.

## Conclusions

In this study, we constructed a novel combination model using different types of compound features that had high performance for rat CL prediction. Although this combination model was effective for rat CL, it may be applicable to other pharmacokinetic parameters, toxicity, and pharmacological activity. This combination QSAR method enables virtual screening from a library of compounds and accelerates drug discovery. Furthermore, this model is expected to enable the construction of a prediction model that outperforms previous models for drug discovery as well as other compound-based targets. Therefore, it is expected to be widely applicable in various fields when prediction performance is an issue.

## Experimental Section

### Experimental Data

All procedures for the animal experiments were approved by the Animal Ethics Committee of Japan Tobacco Inc., Central Pharmaceutical Research Institute. The CL of compounds that were synthesized in multiple in-house projects and subjected to rat PK was obtained from the in-house database. All results were obtained after the intravenous administration of compounds to rats at doses of 0.03–10.0 mg/kg. CL was estimated after the intravenous administration by the noncompartmental analysis of individual plasma concentration–time profiles. In this study, the results of the CL of 1545 in-house compounds were used to construct the prediction models. The threshold for the classification of CL values was 1 L/h/kg, which is approximately 30% of the hepatic blood flow rate in rats.^32^ This threshold is equivalent to approximately 70% for the bioavailability (BA) of compounds eliminated by the liver alone, assuming that the fraction absorbed (Fa) and fraction intestinal availability (Fg) are 1. McIntyre *et al*. developed similar prediction models, but they used 70% of hepatic blood flow as their threshold.^20^ This is equivalent to 30% BA when FaFg = 1. However, it is difficult to obtain a compound with FaFg = 1 early in drug discovery, and the BA is easily likely to be below 30%. Therefore, in this study, the threshold was set at 1 L/h/kg, which is equivalent to 70% BA.

### Calculation of Molecular Descriptors

The structural data of compounds for water molecules and counter ions were eliminated by the processing of disposal salts. Subsequently, the 3D structure of each compound was optimized using “Rebuild 3D” and the force field calculations (amber-10: EHT) were conducted in MOE version 2019.0102 (MOLSIS Inc., Tokyo, Japan). Structural descriptors were calculated employing MOE, alvaDesc (1.0.16) (Alvascience srl, Lecco, Italy), and ADMET Predictor (9.5.0.16) (Simulations Plus, New York, NY, USA). At the time of descriptor generation, descriptors of string type were removed in ADMET Predictor and descriptors of variance 0 were removed in alvaDesc. Overall, 4795 descriptors were selected for further analysis.

### Separation of Compounds into Training and Test Sets and Their Verification by Chemical Space Analysis

After applying stratified random sampling, the compounds in the dataset were separated randomly into a training set and test set at a ratio of 4:1 (Table 5). To investigate the chemical space, 11 molecular parameters were used as reported previously with JMP Pro software 14.3.0 (SAS Institute Inc., Cary, NC, USA) PCA.^21^ The parameters included molecular weight, SlogP (log octanol/water partition coefficient), topological polar surface area (TPSA), h_logD [octanol/water distribution coefficient (pH = 7)], h_pKa [acidity (pH = 7)], h_pKb [basicity (pH = 7)], a_acc (number of H-bond acceptor atoms), a_don (number of H-bond donor atoms), a_aro (number of aromatic atoms), b_ar (number of aromatic bonds), and b_rotN (number of rotatable bonds). The principal components were calculated from 1 to 3.

### Construction of Rat CL Models Based on Molecular Descriptors

**Figure 2:**
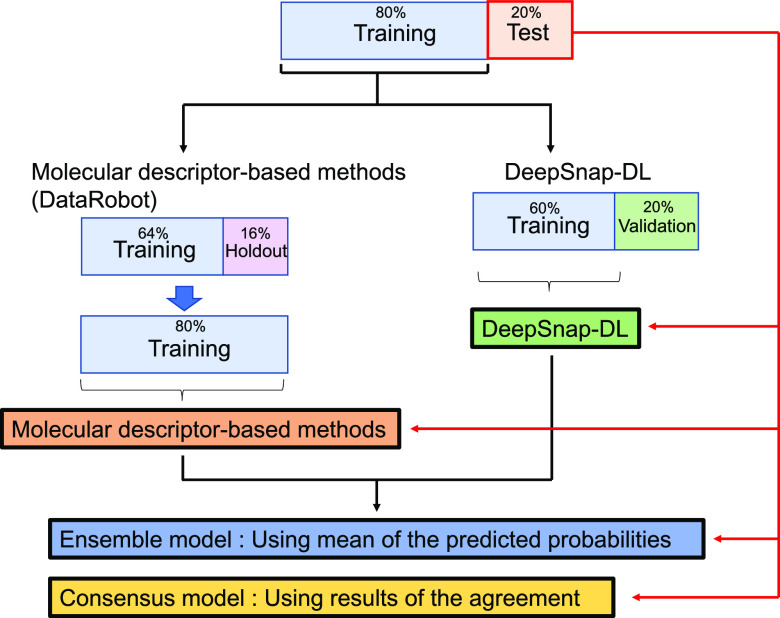
Flowchart of the modeling process for rat CL prediction. For modeling, the 80% training dataset and 20% test dataset were set. The 80% dataset was used to construct prediction models using the molecular descriptor-based method by DataRobot and DeepSnap-DL. Ensemble and consensus models were constructed using the molecular descriptor-based method and DeepSnap-DL. The evaluation metrics of each prediction model were calculated using test sets. DeepSnap DL: DeepSnap Deep Learning.

Model construction based on 4795 molecular descriptors was performed using DataRobot (SaaS, DataRobot, Tokyo, Japan). All analyses were conducted from 29 July 2020 to 31 July 2020. DataRobot automatically performs a modeling competition in which a wide selection of algorithm and data preprocessing techniques compete with one another as reported previously.^33,34^ Prior to training, 20% of the training dataset was randomly selected as the holdout and excluded from training. Five-fold cross-validation was implemented and the partitions were determined with stratified sampling. After the selection of models based on logloss scores of internal validation, molecular descriptors were selected from 4795 molecular descriptors to 100 using the permutation importance. Using these 100 selected molecular descriptors, over 40 models were created and random forest was selected based on validation results as the final algorithm. Following logloss scores of internal validation, the best model was constructed using 100% of the training data. This final model was used to calculate the prediction accuracy of the test sets (Figure 2).

### DeepSnap

3D structures were saved in an SDF file format after “Rebuild 3D” (refer to Calculation of Molecular Descriptors). The 3D chemical structures were depicted as 3D ball-and-stick models with different colors corresponding to different atoms by Jmol, open-source Java viewer software for the 3D molecular modeling of chemical structures, as previously reported.^11−17^ The 3D chemical structures were captured automatically as snapshots with user-defined angle increments with respect to the *x*, *y*, and *z* axes. In this study, a four-angle increment was used: (65°, 65°, 65°), (85°, 85°, 85°), (105°, 105°, 105°), and (145°, 145°, 145°). Other parameters for the DeepSnap depiction process were set based on previous studies^11−17^ as follows: image pixel size, 256 × 256; molecule number per SDF file to split into, 100; zoom factor (%), 100; atom size for van der Waals radius (%), 23; bond radius (mÅ), 15; minimum bond distance, 0.4; bond tolerance, 0.8. The snapshots were saved as 256 × 256 pixel-resolution PNG files (RGB). These were divided into three types of datasets [DeepSnap (Training), DeepSnap (Validation), and test] as described below. After sorting based on CL, training datasets were separated randomly into DeepSnap (Training) or DeepSnap (Validation) at a ratio of 3:1.

### Deep Learning

All the two-dimensional (2D) PNG images produced by DeepSnap were resized by utilizing NVIDIA DL GPU Training System (DIGITS) version 6.0.0 software (NVIDIA, Santa Clara, CA, USA) on four-GPU systems, Te2D sla-V100 (32 GB), with a resolution of 256 × 256 pixels as input data, as previously reported.^11−17^ To rapidly train and fine-tune the highly accurate Convolutional Neural Network (CNN) using the input DeepSnap (Training) and DeepSnap (Validation) datasets based on the image classification and by building the pretrained prediction model, we used a pretrained open-source DL model, Caffe,^35^ and open-source software on the Ubuntu distribution 16.04LTS. In this study, the deep CNN architecture was GoogLeNet and Adam was used for optimization. In the DeepSnap-DL method, the prediction models were constructed by DeepSnap (Training) datasets using 15–300 epochs with 1 snapshot interval in each epoch, 1 validation interval in each epoch, 1 random seed, a learning rate of 0.0000001–0.001, and a batch size of default in DIGITS. Among the epochs, the lowest loss value in the DeepSnap (Validation) datasets, which is the error rate between the results obtained from the DeepSnap (Validation) datasets and the corresponding labeled dataset, was selected for the subsequent examination of prediction using the test set. The probability of the prediction results with the lowest minimum loss [DeepSnap (Validation)] value was analyzed. The probabilities for each image of one molecule captured at different angles with respect to the *x*, *y*, and *z* axes using DeepSnap-DL were calculated. The medians of each of these predicted values were used as the representative values for target molecules as previously reported.^11−17^

### Combination DeepSnap-DL and Conventional ML

In this study, we investigated the combination of DeepSnap-DL and conventional ML using two methods. The first method was the average of the prediction probabilities. The prediction probabilities obtained by DeepSnap-DL and conventional ML were averaged, and this average value was used as the prediction probability of the new prediction model (ensemble model) (Figure 2). The second method used a prediction model that adopted the results of the agreement between DeepSnap-DL and conventional ML (consensus model) (Figure 2).

### Evaluation of the Predictive Model

The performance of each model in predicting rat CL was evaluated in terms of the following metrics: AUC, BAC, ACC, sensitivity, specificity, F-measure, precision, recall, and MCC calculated using KNIME (4.1.4) (KNIME, Konstanz, Germany). These performance metrics were defined as follows:
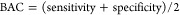

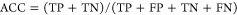

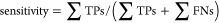

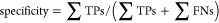

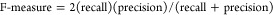

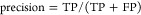

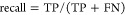

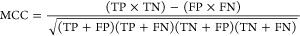
where TP, FN, TN, and FP denote true positive, false negative, true negative, and false positive, respectively. To determine the optimal cutoff point for the definition of TP, FN, TN, and FP, the method of maximizing sensitivity (1 – specificity), termed the Youden index,^36,37^ was used. The index has a value ranging from 0 to 1, where 1 represents over 1 L/h/kg and 0 represents less than 1 L/h/kg for rat CL.

## Supporting Information Available

Physicochemical property distribution of compounds (*n* = 1545) (Figure S1); partition patterns for the model (Figure S2) (PDF)
One hundred molecular descriptors selected by random forest (Table S1); internal validation results using molecular descriptor-based methods (Table S2); prediction performances with 145° on DeepSnap-Deep Learning (Table S3a); prediction performances with 105° on DeepSnap-Deep Learning (Table S3b); prediction performances with 85° on DeepSnap-Deep Learning (Table S3c); prediction performances with 65° on DeepSnap-Deep Learning (Table S3d); details of 11 representative descriptors (Table S4); prediction performances at different partition pattern 1 (Table S5a); prediction performances at different partition pattern 2 (Table S5b); prediction performances at different partition pattern 3 (Table S5c); prediction performances at different partition pattern 4 (Table S5d) (XLSX)

## Notes

The manuscript was written through contributions of all authors. All authors have given approval to the final version of the manuscript. All authors contributed to the study conception and design. Data collection was performed by H.M. Y.U., H.M., and Y.N. developed the QSAR models with experimental data. The first draft of the manuscript was written by H.M. and all authors commented on previous versions of the manuscript.

## ABBREVIATIONS

QSAR: quantitative structure–activity relationship
ADME: absorption, distribution, metabolism, and excretion
PK: pharmacokinetics
CL: clearance
Vd: volume of distribution
ML: machine learning
DL: Deep Learning
DeepSnap-DL: DeepSnap and Deep Learning
CAR: constitutive androstane receptor
AhR: aryl hydrocarbon receptor
ACC: accuracy
AUC: area under the curve
PCA: principal component analysis
BAC: balanced accuracy
MCC: Matthews correlation coefficient
AVG Blender: average Blender
ENET blender: Elastic-Net Blender
MD-based method: molecular descriptor-based method
MOE: Molecular Operating Environment
combi: combinatorial
BA: bioavailability
Fa: fraction absorbed
Fg: fraction intestinal availability
SlogP: log octanol/water partition coefficient
TPSA: topological polar surface area
h_logD: octanol/water distribution coefficient
a_acc: number of H-bond acceptor atoms
a_don: number of H-bond donor atoms
a_aro: number of aromatic atoms
b_ar: number of aromatic bonds
b_rotN: number of rotatable bonds
2D: two-dimensional
DIGITS: NVIDIA DL GPU Training System
CNN: Convolutional Neural Network
