# Supplementary material for: Prediction Model of Clearance by a Novel Quantitative Structure–Activity Relationship Approach, Combination DeepSnap-Deep Learning and Conventional Machine Learning
Source: ACS Omega. 2021 Sep 1;6(36):23570–7. doi: 10.1021/acsomega.1c03689 (PMC8444299; doi:10.1021/acsomega.1c03689)
Supplement: Supplementary file 1 — ao1c03689_si_001.pdf [file ao1c03689_si_001.pdf]

Prediction model of clearance by a novel QSAR approach, combination DeepSnap-Deep Learning and conventional machine learning

Hideaki Mamada[1,2], Yukihiro Nomura[2], Yoshihiro Uesawa[1]*

[1]Department of Medical Molecular Informatics, Meiji Pharmaceutical University, 2-522-1, Noshio, Kiyose-shi, Tokyo 204-858, Japan

[2]Drug Metabolism and Pharmacokinetics Research Laboratories, Central Pharmaceutical Research Institute, Japan Tobacco Inc., 1-1, Murasaki-cho, Takatsuki, Osaka, 569-1125, Japan.

*Corresponding author.

Tel.: +81-42-495-8983; FAX: +81-42-495-8983;

E-mail address: uesawa@my-pharm.ac.jp (Y.U.)

Supporting Information 1

Supporting Table (file type; Excel)

Tabular. Table S1. 100 molecular descriptors selected by randomforest

Tabular. Table S2. The internal validation results using molecular descriptor–based methods

Tabular. Table S3a. Prediction performances with 145° on the DeepSnap-Deep Learning

Tabular. Table S3b. Prediction performances with 105° on the DeepSnap-Deep Learning

Tabular. Table S3c. Prediction performances with 85° on the DeepSnap-Deep Learning

Tabular. Table S3d. Prediction performances with 65° on the DeepSnap-Deep Learning

Tabular. Table S4a. Prediction performances at different partition pattern 1

Tabular. Table S4b. Prediction performances at different partition pattern 2

Tabular. Table S4c. Prediction performances at different partition pattern 3

Tabular. Table S4d. Prediction performances at different partition pattern 4

Supporting Information 2

Supporting Tables and Figures (Figures S1, S2) (file type; WORD)

Figure S1. Physicochemical property distribution of compounds (n = 1545).

Figure S2. Partition patterns for the model

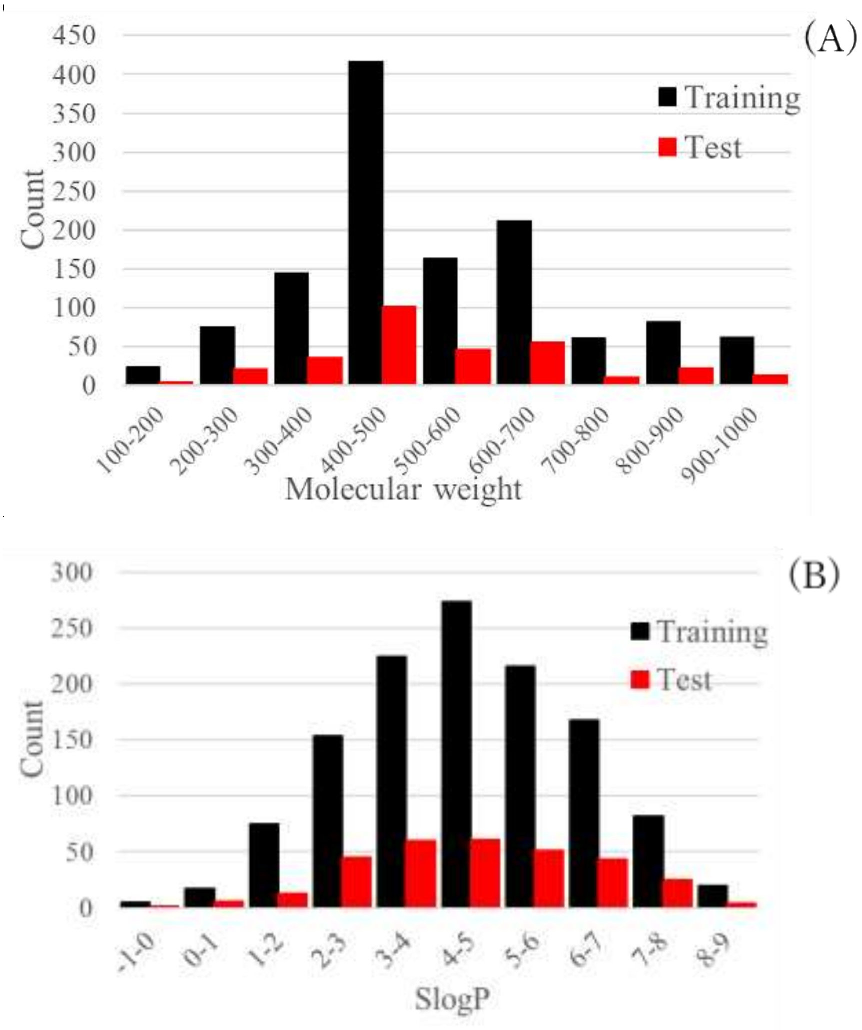

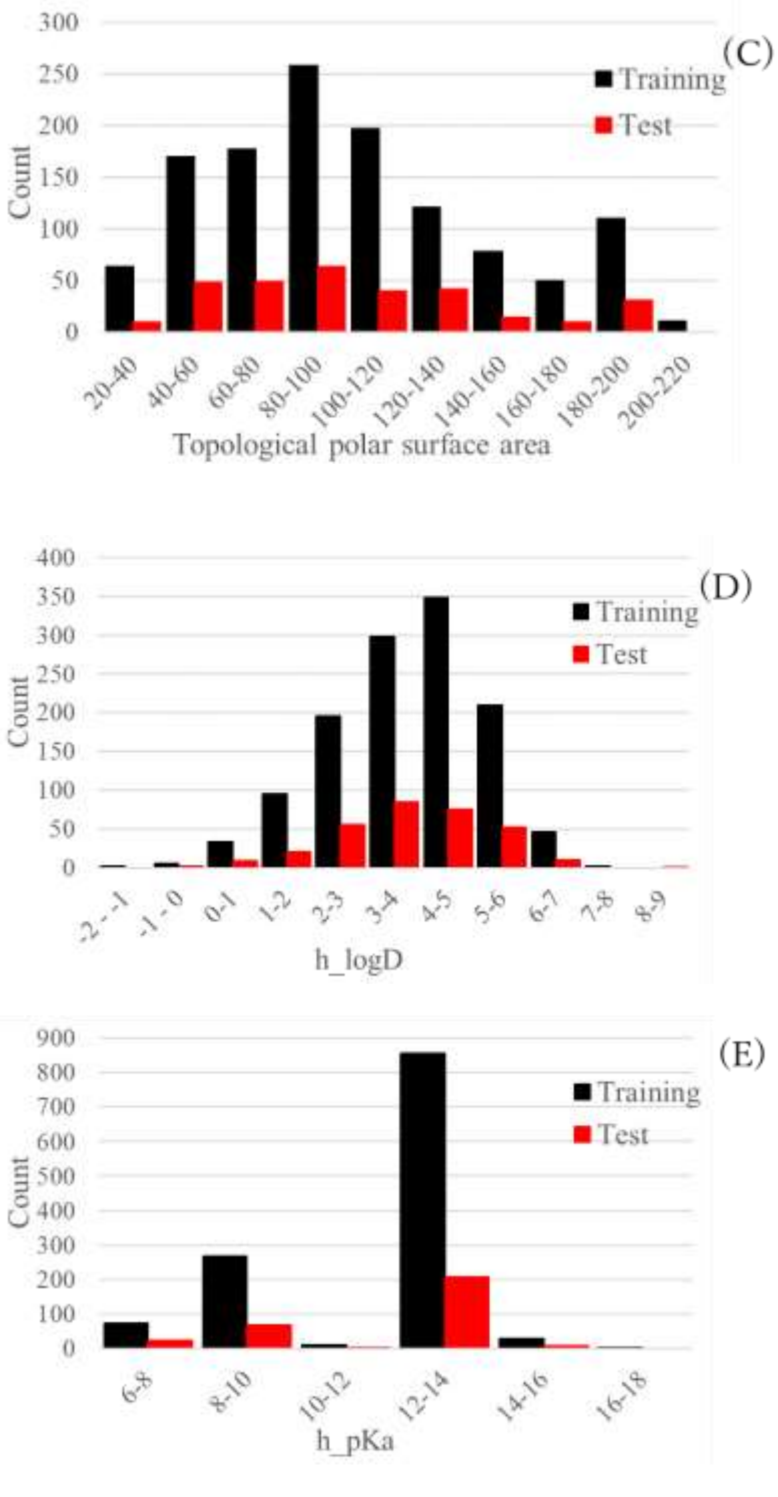
(C)
Training
Test
Count
300
250
200
150
100
50
0
20-40
40-60
60-80
80-100
100-120
120-140
140-160
160-180
180-200
200-220
Topological polar surface area
(D)
Training
Test
Count
400
350
300
250
200
150
100
50
0
-2 - -1
-1 - 0
0-1
1-2
2-3
3-4
4-5
5-6
6-7
7-8
8-9
h_logD
(E)
Training
Test
Count
900
800
700
600
500
400
300
200
100
0
6-8
8-10
10-12
12-14
14-16
16-18
h_pKa

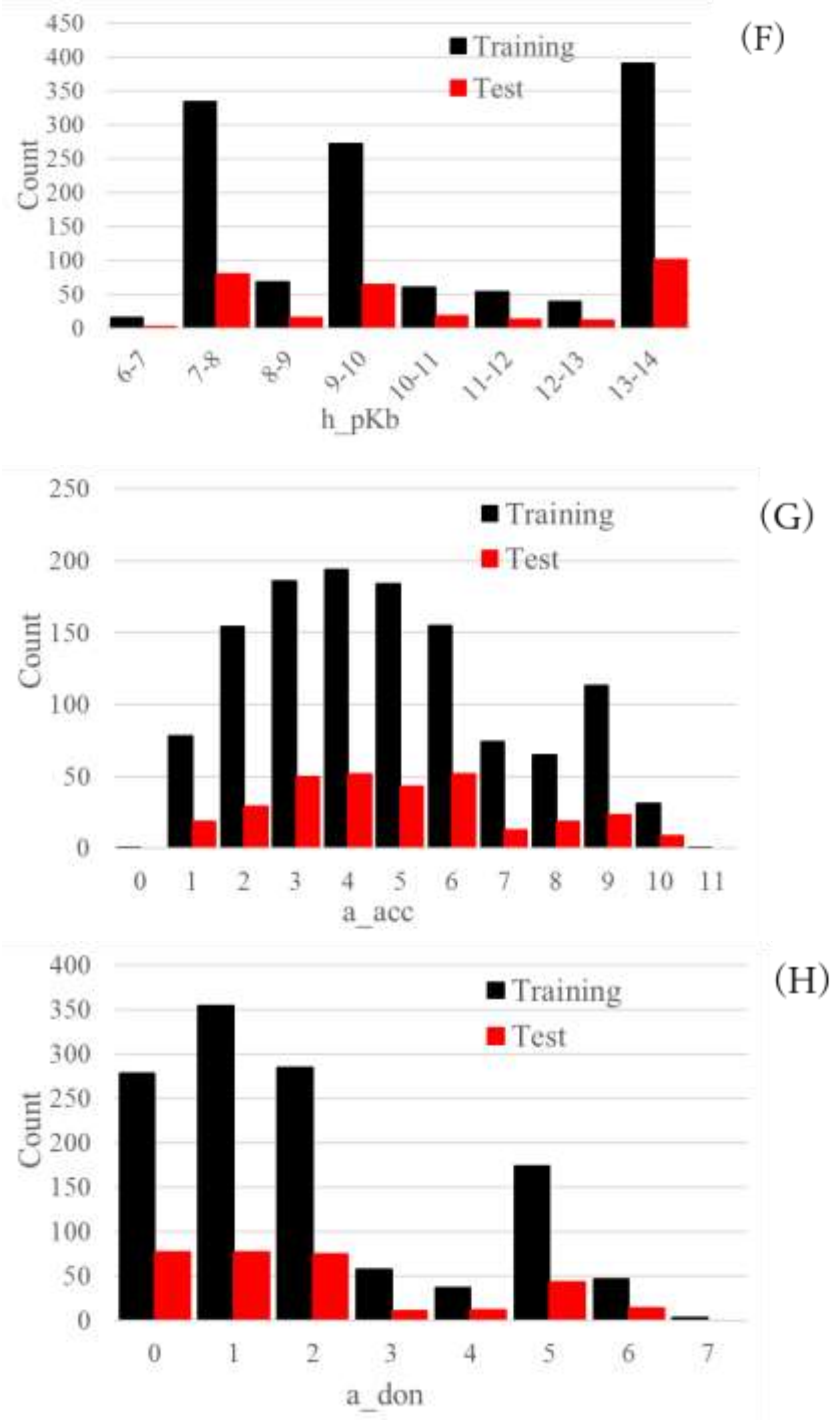
(F)
Training
Test
Count
0
50
100
150
200
250
300
350
400
450
6-7
7-8
8-9
9-10
10-11
11-12
12-13
13-14
h_pKb
(G)
Training
Test
Count
0
50
100
150
200
250
0
1
2
3
4
5
6
7
8
9
10
11
a_acc
(H)
Training
Test
Count
0
50
100
150
200
250
300
350
400
0
1
2
3
4
5
6
7
a_don

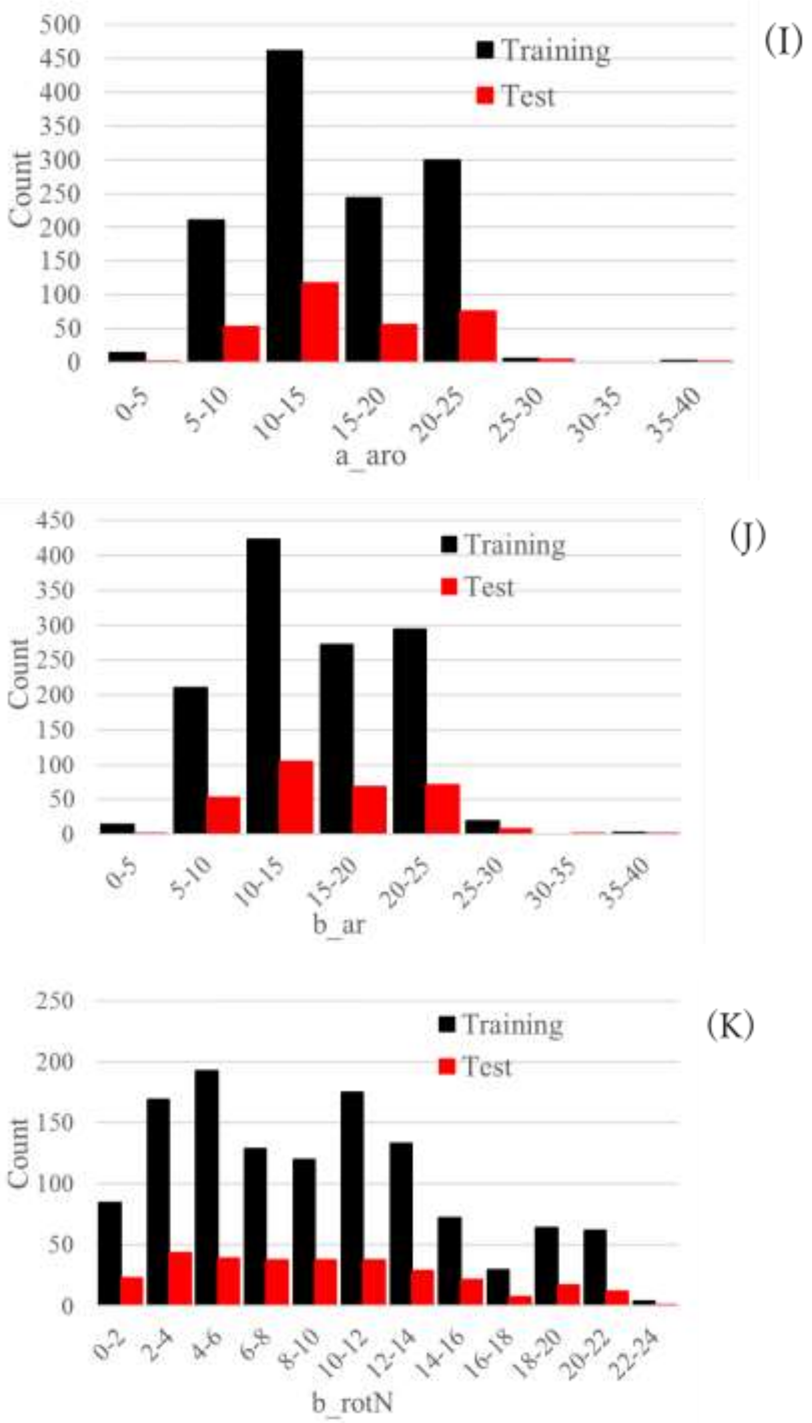
(I)
Training
Test
Count
500
450
400
350
300
250
200
150
100
50
0
0-5
5-10
10-15
15-20
20-25
25-30
30-35
35-40
a_aro
(J)
Training
Test
Count
450
400
350
300
250
200
150
100
50
0
0-5
5-10
10-15
15-20
20-25
25-30
30-35
35-40
b_ar
(K)
Training
Test
Count
250
200
150
100
50
0
0-2
2-4
4-6
6-8
8-10
10-12
12-14
14-16
16-18
18-20
20-22
22-24
b_rotN

Figure S1. Physicochemical property distribution of compounds (n = 1545).

Each physicochemical property distribution represents the molecular weight (A), SlogP (log octanol/water partition coefficient) (B), topological polar surface area (C), h_logD (octanol/water distribution coefficient [pH = 7]) (D), h_pKa (acidity [pH = 7]) (E), h_pKb (basicity [pH = 7]) (F), a_acc (number of H-bond acceptor atoms) (G), a_don (number of H-bond donor atoms) (H), a_aro (number of aromatic atoms) (I), b_ar (number of aromatic bonds) (J), and b_rotN (number of rotatable bonds) (K). Count represents the number of compounds included in each analysis. Black bars represent the training set (n = 1236) and red bars represent the test set (n = 309).

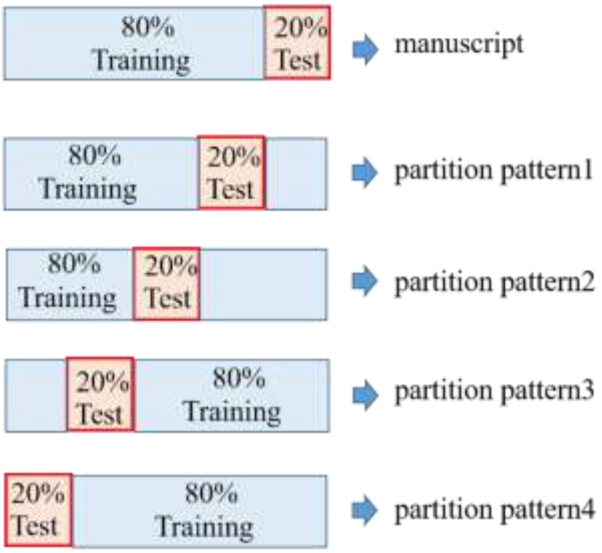

Figure S2. Partition patterns for the model.

One representative result from five split patterns is shown in this manuscript. Other patterns of results are shown in Table S5.
